# Multiple intra-abdominal fungal granulomas caused by *Scedosporium apiospermum* effectively treated with voriconazole in a Golden Retriever

**DOI:** 10.1016/j.mmcr.2023.100611

**Published:** 2023-09-28

**Authors:** Aritada Yoshimura, Ryuji Fukushima, Masaki Michishita, Miki Omura, Koichi Makimura, Daigo Azakami

**Affiliations:** a Animal Medical Center, Tokyo University of Agriculture and Technology, 3-5-8 Saiwaicho, Fuchu, Tokyo, 183-8509, Japan; b Department of Veterinary Pathology, Nippon Veterinary and Life Science University, 1-7-1 Kyonancho, Musashino, Tokyo, 180-8602, Japan; c MycoLabo Inc, #101, 6-6-5-4 Shimorenjaku, Mitaka, Tokyo, 181-0013, Japan; d Institute of Medical Mycology, Teikyo University, 359 Otsuka, Hachioji, Tokyo, 192-0395, Japan; e Laboratory of Veterinary Clinical Oncology, Tokyo University of Agriculture and Technology, 3-5-8 Saiwaicho, Fuchu, Tokyo, 183-8509, Japan

**Keywords:** Dog, Fungal granuloma, Fungal infection, Scedosporium apiospermum, Voriconazole

## Abstract

*Scedosporium apiospermum* is a saprophytic filamentous fungus that is pathogenic to dogs. This report describes a case of *S. apiospermum* infection that caused multiple large peritoneal fungal granulomas in a dog with a history of jejunojejunostomy. The lesions were firmly attached to multiple organs and could not be surgically removed. In such cases, no precedent for the response to the treatment of this disease exists, and all affected dogs have died. This is the first report of an effective medical treatment for multiple intra-abdominal fungal granulomas using voriconazole.

## 1 Introduction

Fungal infections in the intra-abdominal cavity are rare in dogs and usually form peritoneal chronic pyogenic granulomas [[1], [2], [3], [4]]. This lesion is progressive, and causing a wide variety of clinical manifestations related to chronic inflammation or compression of adjacent organs [[1], [2], [3], [4]]. The disease is characterized by a low cure rate using antifungal drugs [1,5]. Therefore, if the lesion cannot be surgically resected, intra-abdominal fungal granuloma has a poor outcome [1,2]. Several fungal species have been reported to cause intra-abdominal fungal granulomas in dogs [[1], [2], [3], [4]]. Among them, *Scedosporium apiospermum* is a representative fungus [[6], [7], [8]]. Lesions caused by this fungus usually grow to enormous size and involve multiple abdominal organs, making them difficult to remove surgically [[6], [7], [8]]. In such cases, no dogs have responded to treatment, which could have served as a precedent; all affected dogs have died [[6], [7], [8]]. Therefore, an appropriate treatment for this disease in dogs has not yet been identified.

In the present study, we isolated *S. apiospermum* from multiple large peritoneal masses in living dog that were difficult to remove surgically due to adhesions. Furthermore, based on the results of antifungal susceptibility testing, we selected medical treatment with voriconazole, which resulted in a favorable clinical course. This case report is the first to describe the diagnostic and medical treatment processes for multiple intra-abdominal fungal granulomas caused by *S. apiospermum* fungus in dogs.

## 2 Case presentation

A 6-year-old castrated male Golden Retriever weighing 10.4 kg, with a body condition score of 2/5 was confirmed to have multiple large masses in the abdominal cavity. The dog was referred to the Animal Medical Center of the Tokyo University of Agriculture and Technology in February 2023 for further examination and treatment [day 0].

**Fig. 1:**
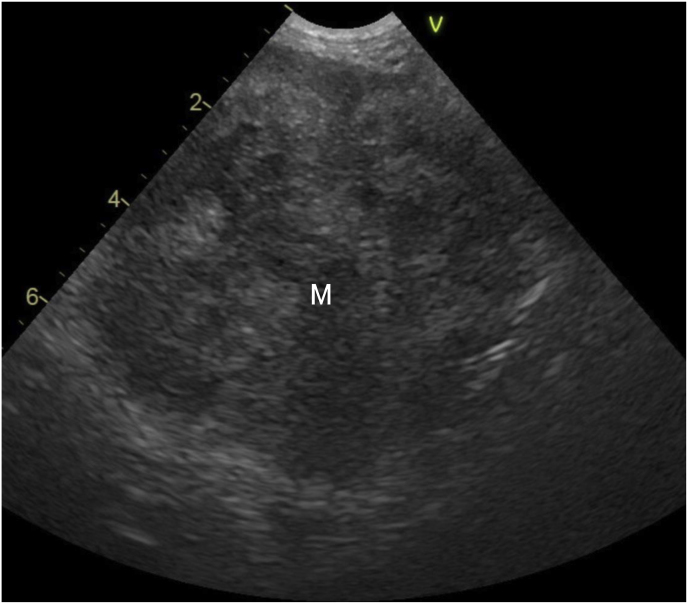
Transverse ultrasonographic image of a representative peritoneal mass in the caudal abdominal cavity. The mass (M) is delineated, and the internal echo displays heterogeneously mixed echogenicity.

**Fig. 2:**
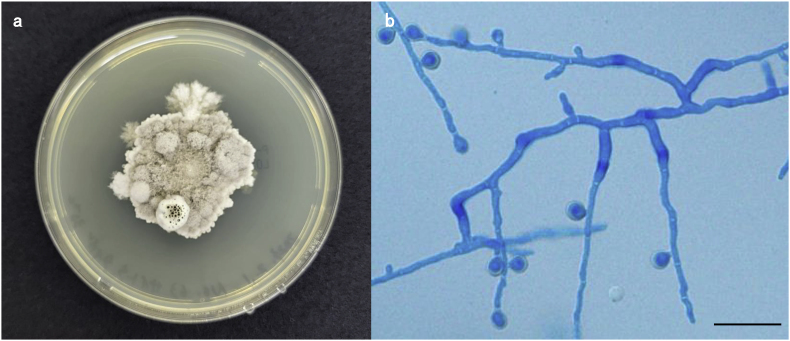
Pure culture of *S. apiospermum* growing on potato dextrose agar. (a) Cottony and wooly colonies with gray-brown centers and gray-white irregular margins were observed after 7 days of culture (This image is at 30th day of culture). (b) Note septate hyphae and a round to oval conidia on a conidiophore extending from the sidewalls (Slide culture preparation stained by lactophenol cotton blue). Bar = 20 μm.

The owner reported that the dog had decreased activity and a loss of appetite. The dog was kept both indoors and outdoors and had undergone jejunojejunostomy due to foreign body ingestion two years earlier. In a follow-up interview on another day, the owner reported that the dog was left free in the yard during the day and had a habit of eating the soil and grass that grew there. The dog had a body temperature of 39.4 °C, a heart rate of 110 beats/min, and a respiratory rate of 42 beats/min. A complete blood count displayed mild increases in the total white blood cell count (21,120/μL). Serum biochemistry exhibited an increase in the levels of total proteins (TP; 8.5 g/dl, reference value 5.0–7.2 g/dl) and C-reactive protein (CRP; >17 mg/dl, reference value < 1.0 mg/dl). Abdominal ultrasonography revealed multiple large spherical masses with a long axis of 30–65 mm and a short axis of 25–65 mm throughout the abdominal cavity. The outlines of these masses were clear and the interior displayed heterogeneously mixed echogenicity (Fig. 1). Fine-needle aspiration (FNA) of the masses revealed numerous neutrophils, macrophages, and septate hyphae. No bacteria were detected in the masses. The FNA samples were mailed to a commercial fungus testing laboratory (MycoLabo Inc., Tokyo, Japan) and Institute of Medical Mycology of Teikyo University for culture and drug susceptibility testing. The sample was inoculated onto potato dextrose agar and the plate was incubated at 35 °C. Fungal growth was observed after 24 h of incubation. Fluffy colonies with gray-brown centers and gray-white margins were observed after a week of culture (Fig. 2a). Light microscopy of the mycological preparations obtained by slide culture (stained with lactophenol cotton blue) revealed septate hyphae and a single circular or oval spore on a conidiophore extending from the lateral wall (Fig. 2b). Based on the aforementioned morphological features, the fungus was presumed to belong to the *Scedosporium* genus. Genetic analyses were performed to identify the species. The fungus was identified as *S. apiospermum* based on the nucleotide sequence of the internal transcribed spacer region of the nuclear ribosomal deoxyribonucleic acid. Drug susceptibility testing revealed high minimum inhibitory concentration (MIC) values for oral antifungal drugs commonly used in veterinary medicine, such as itraconazole (ITCZ), fluconazole (FLCZ), and flucytosine, indicating that the fungus was strongly resistant to these drugs (Table 1). In contrast, the fungus was relatively susceptible to voriconazole (VRCZ), with a moderate MIC value (1 g/mL) (Table 1). However, this result was insufficient to ensure the efficacy of medical treatment with antifungal drugs. Therefore, computed tomography (CT) and laparotomy were performed under general anesthesia for the surgical removal of the masses [day 16].

**Table 1:** Antifungal susceptibility test results presented as minimal inhibitory concentration (MIC).

Oral antifungal agent	MIC (μg/ml)
5-Flucytosine	>64
Amphotericin B	16
Fluconazole	16
Itraconazole	>8
Voriconazole	1

**Fig. 3:**
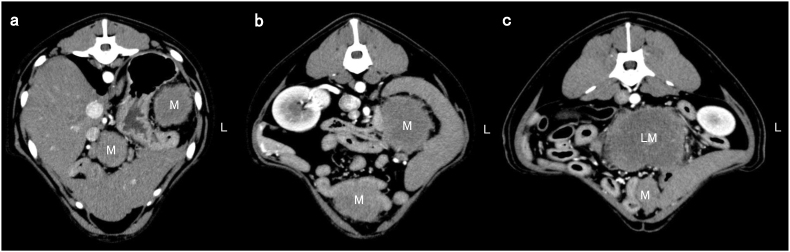
Axial computed tomography image of representative peritoneal masses in the abdominal cavity. (a) Soft tissue masses (M) are observed in the peritoneum near the lesser and greater curvature of the stomach (47 × 36, and 44 × 39 mm, respectively). (b) Similar masses (M) are observed in the splenic hilum and mesenteric regions (53 × 47, and 45 × 36 mm, respectively). (c) The largest mass (LM) is located in mesenteric regions (69 × 55 mm. Same as the mass shown in Fig. 2).

**Fig. 4:**
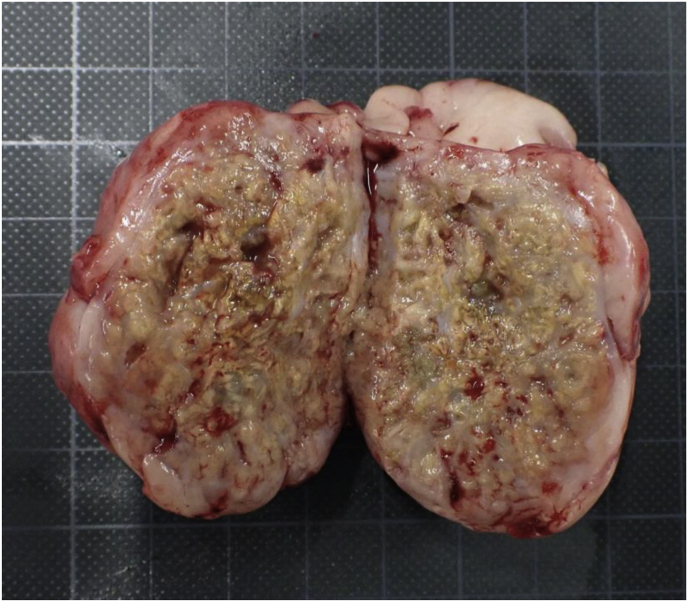
Macroscopic findings of resected fungal granuloma. Numerous yellow-to-white nodules of various sizes are observed inside the lesion.

**Fig. 5:**
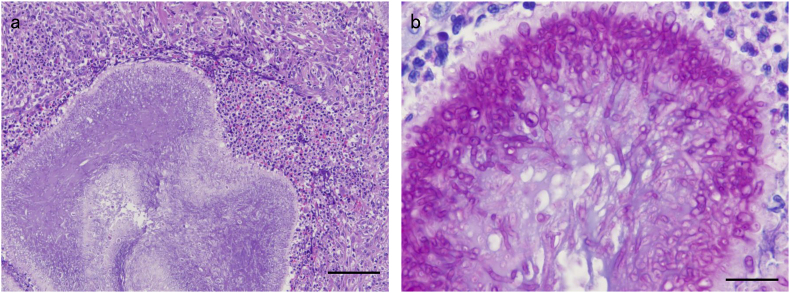
Histopathological findings of resected fungal granuloma. (a) Numerous fungi are observed in the center of the nodule. A high degree of infiltration of neutrophils, macrophages, and epithelioid cells and hyperplasia of collagen fibers are observed around the fungus. Hematoxylin and Eosin stain. Bar = 100 μm. (b) The fungus had septa and was positive for PAS staining. PAS stain. Bar = 20 μm.

Moreover, CT scan revealed a total of nine masses in the peritoneum in the following areas: the lesser and greater curvature regions of the stomach (47 × 36 and 44 × 39 mm, respectively), the splenic hilum region (53 × 47 mm), the mesenteric region (3 pieces, 69 × 55 and 45 × 36, respectively), and just below the white line (3 pieces, 37 × 29, 29 × 20, 16 × 14 mm, respectively) (Fig. 3a–c). The mass did not originate from the abdominal organs. No obvious lesions were observed in other areas. During laparotomy, most masses were firmly attached to the jejunum, pancreas, main portal vein, and other abdominal organs. Therefore, only resectable masses (in the greater curvature of the stomach and just below the white line) were surgically removed. Numerous yellow-to-white nodules of various sizes were observed inside the masses (Fig. 4). Histopathological examination revealed septate hyphae in the center of the nodule, which were surrounded by high infiltration of neutrophils, macrophages, epithelioid cells, and proliferation of collagen fibers (Fig. 5a). Furthermore, the hyphae were positively stained with Periodic acid-Schiff (PAS) and Grocott's stains (Fig. 5b). Based on the findings, the dog was definitively diagnosed with pyogenic granulomatous inflammation caused by a fungal infection, fungal granuloma. Therefore, medical treatment with VRCZ was initiated (at a dose of 5 mg/kg p. o bid) [day 24].

After the initiation of VRCZ administration, the dog gradually regained vigor and appetite. One month after starting oral administration [day 53], normalization of TP (6.5 g/dl) and a decrease in CRP (1.9 μg/dl) were observed. Abdominal ultrasonography displayed that each fungal granuloma had reduced in size by approximately 10–15%. Furthermore, 2 months after starting oral administration [day 83], normalization of CRP (0.3 g/dl) was observed along with a reduction in the size of each fungal granuloma by approximately 25–30% compared to that before initiation of medication. At this point, the clinical signs completely disappeared and weight gain was observed. Furthermore, at present, 4 months after starting oral administration [day 148], each fungal granuloma has reduced in size by approximately 45–50%, and the dog is doing well. Furthermore, no side effects of VRCZ, such as elevated liver enzymes or gastrointestinal symptoms, have been observed. Therefore, we will continue this treatment until the lesions completely resolve.

## 3 Discussion

In humans, the primary route of *S. apiospermum* infection is thought to be direct dissemination into deep tissues associated with penetrating trauma or surgery [5,9]. In this case, fungal granuloma was identified only in the peritoneum and not in the subcutaneous tissue or muscle. This indicated that the fungus was disseminated directly into the intra-abdominal cavity. Although, no history of abdominal cavity trauma was present, the dog had a history of gastrointestinal anastomosis under laparotomy 2 years earlier. Additionally, *S. apiospermum* is a saprophytic filamentous fungus that is widely and commonly isolated from soils in temperate regions, and the growth of the fungus is promoted by manure-enriched environments [5]. The dog was left to roam free in the garden for most of the day and had a habit of eating soil and grass. Therefore, it was highly likely that the dog ingested *S. apiospermum* daily. Based on these facts, we speculated that the fungus most likely disseminated directly into the abdominal cavity with intestinal contents during surgery or the healing process. Another possible route is fungal translocation from the gastrointestinal tract. The host immunocompetence is participated in the establishment of fungal infection. Therefore, temporary immune dysfunction due to surgery, or a congenital one can have contributed to the infection. However, immune function is difficult to assess in dogs, hence the details are unclear.

Other diseases that cause masses in the abdominal cavity include tumors, foreign body granulomas, actinomycetes, and mycobacterium infections [10,11]. In this case, distinguishing between the aforementioned diseases using ultrasonography and CT tomography was difficult. However, FNA cytology confirmed fungal granuloma and a combination of fungal culture and genetic analysis identified the pathogen as *S. apiospermum*. Other pathogenic *Scedosporium* species identified in dogs include *S. prolificans*. Moreover, *S. prolificans* is more resistant to antifungal drugs and prone to systemic dissemination than *S. apiospermum* [5,9]. Therefore, the differentiation of these fungi is clinically important for prognostic prediction. In general, reliable culture identification based on fungal morphological differences is considered difficult to identify species [12]. Therefore, reliable species identification using molecular biological techniques is recommended.

Generally, the first-line treatment for fungal granuloma is surgical excision of the lesion [9]. This is because the lesions surrounded by a thick fibrotic capsule contain large amounts of necrotic tissue and antifungal drugs do not penetrate sufficiently [1,2]. However, in this case, strong adhesions between the lesions and the abdominal organs were observed, making complete removal of these lesions difficult. We selected antifungal therapy for the residual lesions and performed a drug susceptibility test. The fungal strain isolated exhibited strong resistance to ITCZ and low susceptibility to FLCZ, which are frequently used in veterinary medicine. In humans, *S. apiospermum* exhibits strong natural resistance to conventional antifungal drugs [5,9]. Hence, we conclude that the results of this susceptibility test reflect the innate characteristics of *S. apiospermum*. Fortunately, the fungal strain isolated from this dog was susceptible to VRCZ. This was considered a key point for successful treatment in this case. If no effective antifungal drug had been identified, the dog would have had a fatal clinical course, as in previous reports.

In the present case, *S. apiospermum* infected the peritoneum and formed a fungal granuloma, resulting in chronic activity and loss of appetite. Veterinarians should carefully include fungal granuloma caused by *S. apiospermum* in the differential diagnosis when a peritoneal mass is observed. The selection of an appropriate antifungal drug allows medical therapy for this disease. Therefore, VRCZ may be effective against fungal infections.

## Conflict of interest

The authors declare that the research was conducted in the absence of any commercial or financial relationships that could be construed as a potential conflict of interest.
